# Supplementary material for: Testing predictability of disease outbreaks with a simple model of pathogen biogeography
Source: R Soc Open Sci. 2019 Nov 13;6(11):190883. doi: 10.1098/rsos.190883 (PMC6894608; doi:10.1098/rsos.190883)
Supplement: Supplemental Materials [file rsos190883supp1.pdf]

Supplemental materials

Testing predictability of disease outbreaks with a simple model of pathogen biogeography

Tad A. Dallas, Colin J. Carlson, Timothée Poisot

Further detail on the *plug-and-play* model

We present the *plug-and-play* model in the main text as a means to estimate the suitability of a pathogen being found in a given country as a function of 1) year, 2) pathogen community dissimilarity between countries (C_{ij}), 3) pathogen distributional dissimilarity (P_{ij}), and 4) the product of these two values ($C_{ij} \times P_{ij}$). Here, we provide more detail on the modeling approach. Further methodological detail is provided elsewhere (36), as is the application of the approach to estimating potentially missing links in host-parasite networks given host and pathogen trait data (37).

The goal of the modeling approach is to estimate the conditional suitability of a given country-pathogen pair given the set of observed ($y = 1$) and unobserved ($y = 0$) country-pathogen pairs. That is, the suitability of a given pathogen and country pair is given by

$$P(y = 1|x) = \frac{f_1(x)P(y = 1)}{f(x)}$$

where f_1 is the density of feature set $x_{i,j}$ given that $y_{i,j} = 1$, $P(y = 1)$, and f is the density of all possible combined feature sets. The feature set $x_{i,j}$ here consists of the four variables discussed above, relating to the dissimilarity of pathogen communities in countries and pathogen distributions, in addition to the year for which the dissimilarity values were estimated. We estimate suitability of a country-

pathogen pair as the ratio of these two densities

$$q = \frac{f_1}{f}$$

The *plug-and-play* model estimates f_1 and f separately to estimate q, using the kernel density estimator `npudens` in the `np` package (38) and the "normal-reference" bandwidth.

Effect of rolling window size

The size of the rolling window we used for model training prior to prediction could influence model performance. To examine this possibility, we used a rolling window of 7 years (compared to the 4 year window used in the main text), finding qualitatively similar results when flu was included (Figure S1) or excluded (Figure S2). We explored this further by examining rolling windows of 2, 4, and 6 years (Figure S3), with qualitatively similar findings. For this analysis, we excluded influenza, as we did in the main text.

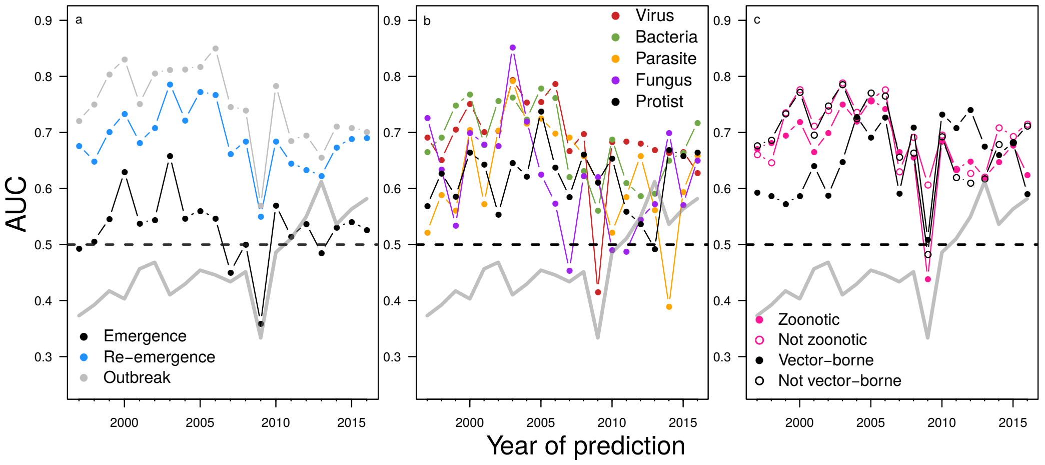

Figure S1: Rolling window size did not strongly influence model performance when considering next year prediction, as a window of 7 years produced qualitatively similar results to the window of 4 years we examine in the main text. The null expectation (grey line) performed consistently worse than our approach.

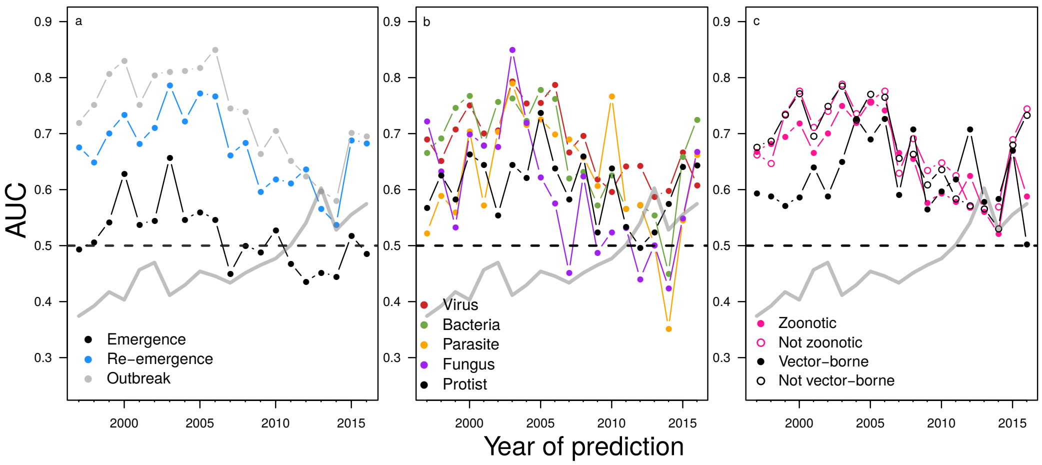

Figure S2: Rolling window size did not strongly influence model performance when considering next year prediction, as a window of 7 years produced qualitatively similar results to the window of 4 years we examine in the main text. The null expectation (grey line) performed consistently worse than our approach.

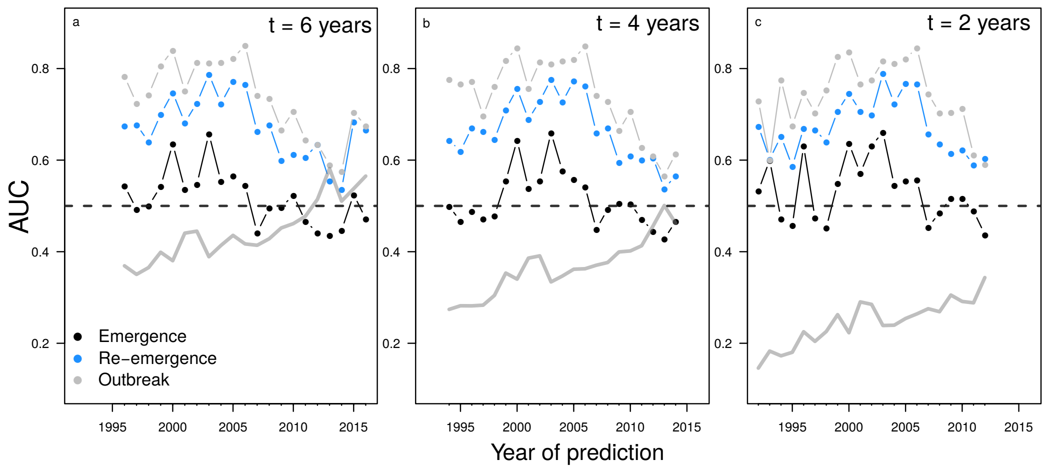

Figure S3: Examining rolling windows of 2, 4, and 6 years provides evidence that rolling window size did not strongly influence model performance. The null expectation (grey line) performed consistently worse than our approach.

Effect of pathogen traits on model hindcasting/forecasting ability

Here, we further explore the effect of pathogen type on model performance when hindcasting or forecasting pathogen outbreak or emergence event suitability. There was no predictable variation in model performance as a function of pathogen type (Figure S4) or whether the pathogen is classified as zoonotic or vector-borne (Figure S5).

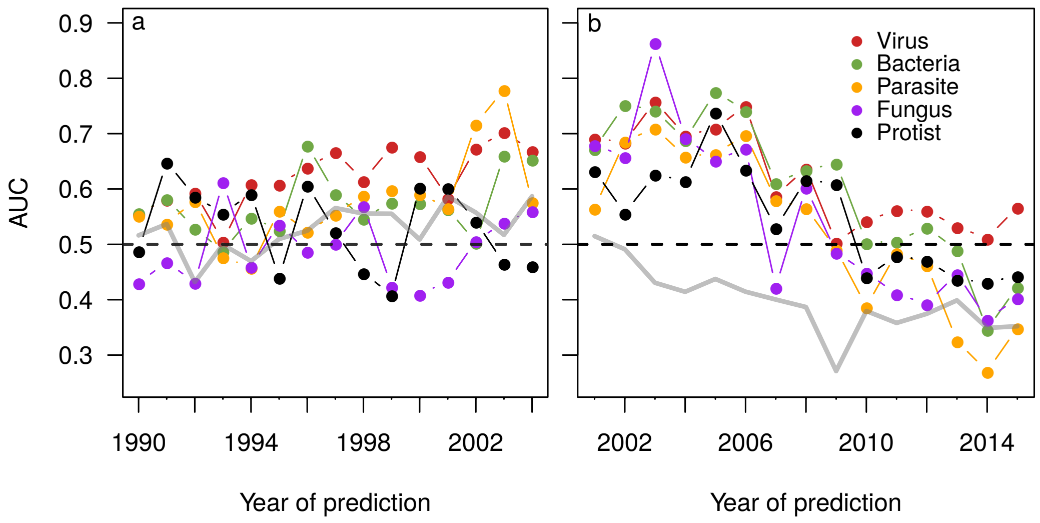

Figure S4: Predictive accuracy decreased when attempting to forecast far into the past or future, independent of pathogen type (grey line corresponds to null model performance). Models were trained on either the period between 2005-2015 (for prediction into the past) or 1990-2000 (for prediction into the future).

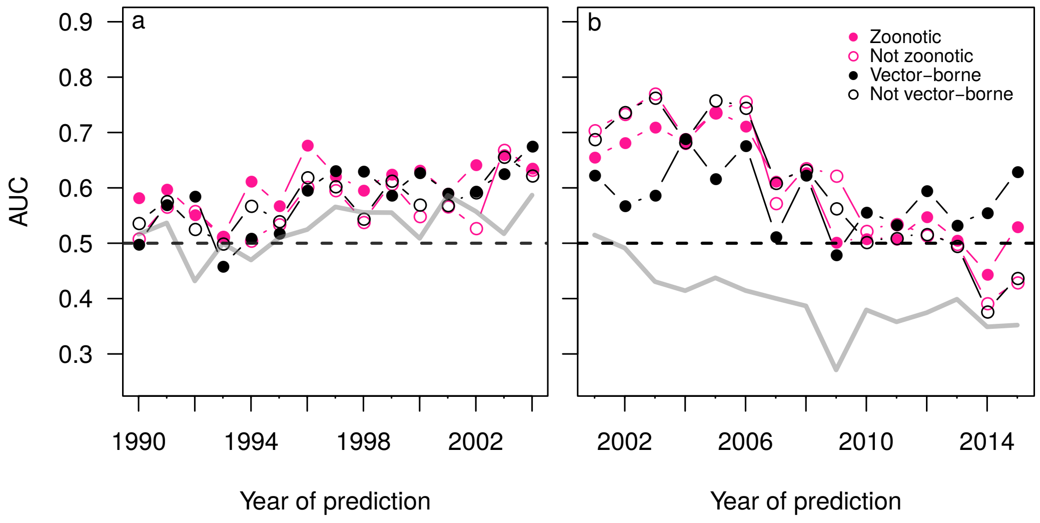

Figure S5: Predictive accuracy decreased when attempting to forecast far into the past or future. This was insensitive to whether the pathogen is considered zoonotic or vector-borne. Models were trained on either the period between 2005-2015 (for prediction into the past) or 1990-2000 (for prediction into the future). The null expectation (grey line) performed consistently worse than our approach.

The effect of including influenza

The 2009 influenza A pandemic fundamentally changed the network of countries and pathogens through the addition of many links to one pathogen (Figure S6). This may be an issue for approaches such as ours, which relies on extracting information from the similarity between pathogens in their distributions among countries, and similarity between countries in their pathogen composition. When the model wasn't expected to predict a pandemic event, the inclusion of influenza did not substantially influence model predictions when trained on differing numbers of years (Figure S7) or when forecasting or hindcasting to different time periods (Figure S8). However, the effect of the 2009 influenza pandemic can be seen in the substantial declines in model performance when attempting to forecast one year ahead after training on a rolling window of 4 years (Figure S9). Interestingly, the exclusion of influenza results in lower mean performance when the model doesn't have data on many years, likely because influenza is widespread and can influence the pathogen and country dissimilarity values used to train the model. However, once sufficient data is provided, model performance with and without influenza is nearly identical.

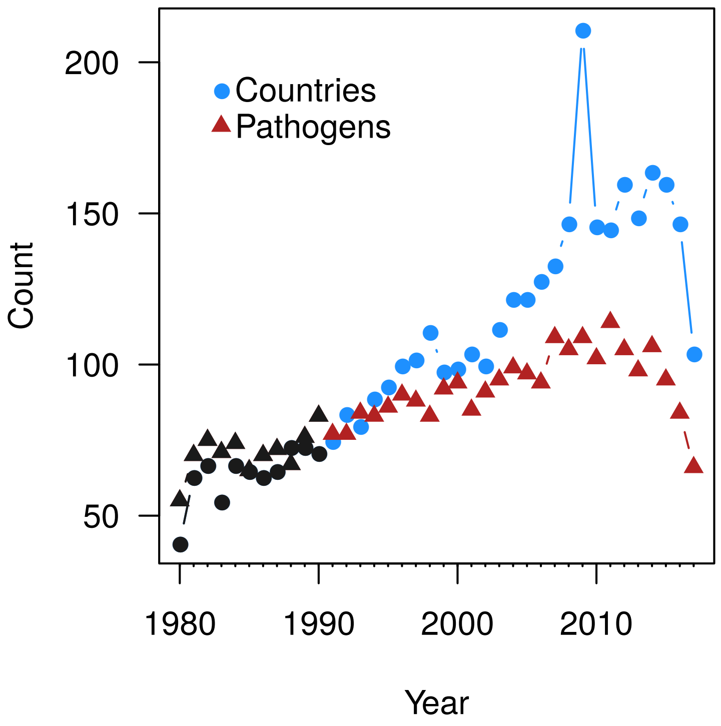

Figure S6: The number of countries with at least one outbreak event and the number of pathogens has increased over time, likely due to more vigilant sampling and description of emerging pathogens in a larger number of countries.

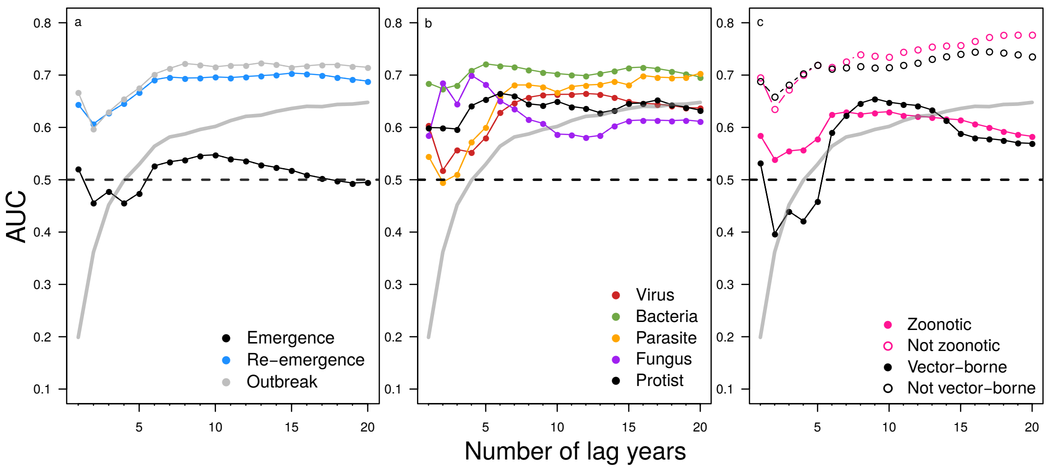

Figure S7: Pathogen events from previous years increased model predictive accuracy after an initial small decrease, suggesting that five years or more of data improves predictions, but accuracy could actually decrease in some data sparse situations where only two or three years of data were available. Performance of the null expectation (grey line) was less than our approach, except when the null was given more than 15 years of previous data.

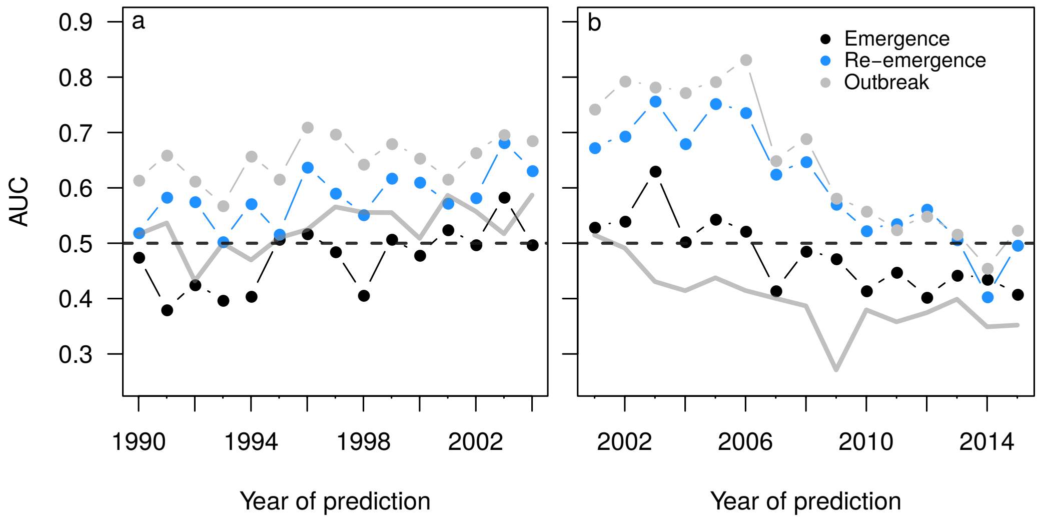

Figure S8: Predictive accuracy decreased when attempting to forecast far into the past or future. Models were trained on either the period between 2005-2015 (for prediction into the past) or 1990-2000 (for prediction into the future). The null expectation (grey line) performed consistently worse than our approach.

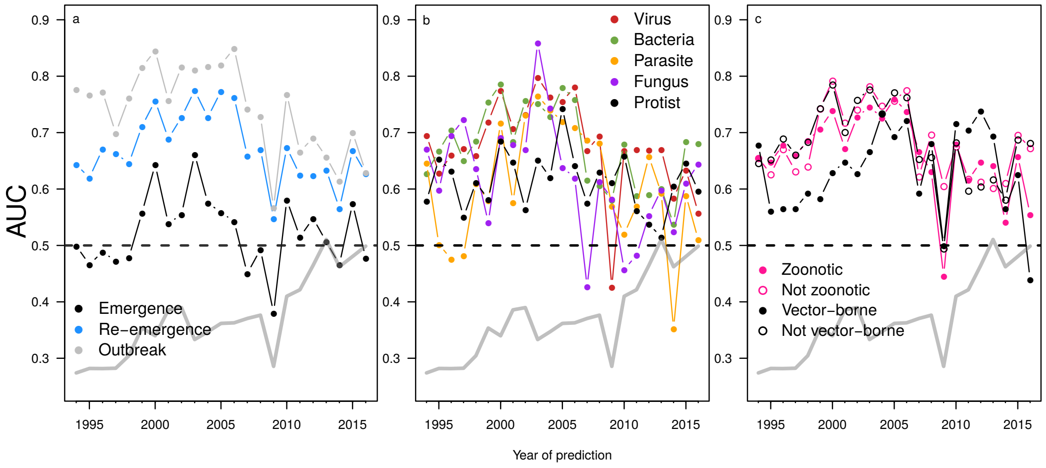

Figure S9: Using a rolling window ($t = 4$ years), we found that predictive accuracy did not increase as a result of enhanced surveillance and data collection of more recent years. The null expectation (grey line) performed consistently worse than our approach.
